# Proof of Concept, Randomized, Placebo-Controlled Study of the Effect of Simvastatin on the Course of Age-Related Macular Degeneration

**DOI:** 10.1371/journal.pone.0083759

**Published:** 2013-12-31

**Authors:** Robyn H. Guymer, Paul N. Baird, Mary Varsamidis, Lucy Busija, Peter N. Dimitrov, Khin Zaw Aung, Galina A. Makeyeva, Andrea J. Richardson, Lyndell Lim, Liubov D. Robman

**Affiliations:** 1 Centre for Eye Research Australia, University of Melbourne, Royal Victorian Eye and Ear Hospital, East Melbourne, Victoria, Australia; 2 Biostatistics Unit, Faculty of Health, Deakin University, Burwood, Victoria, Australia; Medical University Graz, Austria

## Abstract

**Background:**

HMG Co-A reductase inhibitors are ubiquitous in our community yet their potential role in age-related macular degeneration (AMD) remains to be determined.

**Methodology/Principal Findings:**

Objectives: To evaluate the effect of simvastatin on AMD progression and the effect modification by polymorphism in apolipoprotein E (*ApoE*) and complement factor H (*CFH*) genes. Design: A proof of concept double-masked randomized controlled study. Participants: 114 participants aged 53 to 91 years, with either bilateral intermediate AMD or unilateral non-advanced AMD (with advanced AMD in fellow eye), BCVA≥20/60 in at least one eye, and a normal lipid profile. Intervention: Simvastatin 40 mg/day or placebo, allocated 1∶1. Main outcome measures: Progression of AMD either to advanced AMD or in severity of non-advanced AMD. Results. The cumulative AMD progression rates were 70% in the placebo and 54% in the simvastatin group. *Intent to treat* multivariable logistic regression analysis, adjusted for age, sex, smoking and baseline AMD severity, showed a significant 2-fold decrease in the risk of progression in the simvastatin group: OR 0.43 (0.18–0.99), p = 0.047. Post-hoc analysis stratified by baseline AMD severity showed no benefit from treatment in those who had advanced AMD in the fellow eye before enrolment: OR 0.97 (0.27–3.52), p = 0.96, after adjusting for age, sex and smoking. However, there was a significant reduction in the risk of progression in the bilateral intermediate AMD group compared to placebo [adjusted OR 0.23 (0.07–0.75), p = 0.015]. The most prominent effect was observed amongst those who had the *CC* (*Y402H*) *at risk* genotype of the *CFH* gene [OR 0.08 (0.02–0.45), p = 0.004]. No evidence of harm from simvastatin intervention was detected.

**Conclusion/Significance:**

Simvastatin may slow progression of non-advanced AMD, especially for those with the *at risk CFH* genotype *CC* (*Y402H*). Further exploration of the potential use of statins for AMD, with emphasis on genetic subgroups, is warranted.

**Trial Registration:**

Australian New Zealand Clinical Trial Registry (ANZCTR) ACTRN1260500032065

## Introduction

The possible use of HMG Co-A reductase inhibitors, or statins, to slow AMD progression, has been considered for some time. Their pleiotropic actions, such as their lipid-lowering and anti-inflammatory actions, could impact on the underlying pathological changes involved in AMD pathogenesis.[1], [2] An inverse association between the use of statins and AMD development has been reported in a number of retrospective [3]–[6] and prospective [7] studies, including our own,[4] as well as in a meta-analysis of eight studies.[8] However, other studies failed to detect similar associations [9]–[16] or even found a harmful effect of long-term simvastatin intake, with increased hazard rate for developing exudative AMD.[17] The need for a prospective randomized controlled trial (RCT) that could address the potential benefits of statins in AMD was highlighted in recent reviews, including a Cochrane review.[18], [19] Finding a safe and effective intervention to slow progression of AMD becomes more urgent as our population ages and the possibility that one may already exist within our armamentarium would significantly hasten its introduction if it were found to be effective.

Our first objective was to determine if there is any potential efficacy signal of HMG Co-A reductase inhibitor ‘simvastatin’ on the overall progression of AMD, either to advanced disease or to a greater severity of early stage disease. The second aim was to investigate the possible influence of genetic variants of the complement factor H (*CFH*) or apolipoprotein E (*APOE*) genes on efficacy of simvastatin intervention. Our hypotheses were that simvastatin would slow down AMD progression, and that this effect could be more prominent at different AMD stages or in genetically different subgroups. This study also conducted surveillance of potential harm from simvastatin in people whose lipid profile would not trigger the use of lipid-lowering medications for the prevention of cardiovascular disease.

## Materials and Methods

### Study Design

The design and methodology of this study has been described previously.[20] Briefly, this was a proof-of-concept, randomized, placebo-controlled (allocation ratio 1∶1), double-masked, three year study of simvastatin, 40 mg daily, in participants with non-advanced AMD in at least one eye, considered at high risk of progression towards advanced AMD. Participants were recruited from studies on the natural history of AMD or from medical retinal clinics in Melbourne. The study was conducted at the Centre for Eye Research Australia (CERA), University of Melbourne, with the examination sites located at the Royal Victorian Eye and Ear Hospital (RVEEH) and the Caulfield General Medical Centre. The protocol for this trial and supporting CONSORT checklist are available as supporting information; see Checklist S1 and Protocol S1.

### Ethics Statement

The project was approved by the Research and Ethics Committee of the RVEEH, undertaken according to the Helsinki Declaration for the research on humans and registered with the Australian New Zealand Clinical Trials Registry (ACTRN12605000320651, http://www.anzctr.org.au/). Written informed consent was obtained from all participants prior to entry into the study.

### Recruitment

This study was specifically designed to enrol patients at high risk of AMD progression. Eligibility criteria required that participants have at least 1 large druse (>125 um) or extensive intermediate drusen (63–125 um) with pigment change (intermediate AMD)[21] in both eyes, or advanced AMD [choroidal neovascularization (CNV) or geographic atrophy [GA]) in one eye and any non-advanced AMD features in the study eye. A visual acuity of 20/60 or better in the study eye, a blood lipid profile that did not meet the criteria of the National Heart Foundation of Australia guidelines for treatment with a lipid lowering agent [22], [23] and absence of confounding ophthalmological diseases such as glaucoma, diabetic retinopathy or advanced cataract that could interfere with retinal photographic and functional assessments were also required.[20]

### Study Examinations

Prior to randomization, a standard eye examination was performed, including measurement of best corrected visual acuity (BCVA), a dilated slit lamp examination with grading of lens opacities, digital macular photography using a Canon CR6-45NM Non-Mydriatic Retinal Camera (Saitama, Japan) and a variety of retinal visual function tests. Baseline assessment also included questionnaires on demographics, general medical history, dietary intake, medications, ethnic origin, and family history of AMD. Blood samples were collected to test for liver function, lipid profile, C-reactive protein levels, and genetic polymorphisms.

Biannual follow-up examinations were conducted for three years after randomization. At each review visit, participants underwent a full eye examination and blood tests. If clinically indicated, fluorescein angiography was undertaken to exclude/confirm CNV. Participants with confirmed CNV were subsequently managed in the retinal clinic at RVEEH.

### Treatment allocation

Participants were randomly assigned to receive 40 mg of simvastatin or placebo in tablets of identical appearance and taste (prepared by MSD AUSTRALIA [Merck Sharp & Dohme (Australia) Pty Ltd], NSW, Australia). Randomization was performed by a biostatistician using permuted blocks of randomly varying size.[20] The allocation list was stored at a remote site. The study staff, the participants, and data analysts were masked to treatment allocation until the analysis was finalised. The hospital pharmacist packed the medication into identical containers according to the randomization code. The sequentially numbered containers were allocated to the participants by the study coordinator in order of enrolment.

### Compliance and adverse events

Participants who were advised by their treating physician to start cholesterol lowering medication during the course of the study were asked to start 40 mg of simvastatin and were allocated ‘off protocol’ status. Compliance was determined using self-reporting, counting unused tablets and by measuring each subject's lipid profile every 6 months. Liver function tests were conducted at each review. Adverse events were reviewed by a safety monitoring committee with severe adverse events reported to the ethics committee. The trial would be stopped if rates of drug-related adverse events were found to be significantly higher in the active treatment group.

### Assessment of AMD status

Fundus examination and photography were performed at each visit. Digital images of each macula were graded according to the International Classification and Grading System for AMD by two trained graders, masked to treatment allocation.[24] Grading was conducted using the ‘OptoMize PRO’ software from Digital Healthcare Image Management System (Digital Healthcare Ltd (DH), Cambridge, UK). Each macula was graded within a 6000 um diameter grid centred on the fovea for type, size, location, number, centrality and area covered by AMD features. Thus, drusen type (intermediate, soft distinct or soft indistinct), number (1–9, 10–19, 20 or more), size (>63 µ, >125 µ, >175 µ, >250 µ), centrality (fovea, central, middle, outer subfields or outside the grid), and area covered (<10%, <25%, <50%, >50% of the areas delineated by the central, middle and outer circles of the grid) were determined. For pigment changes, differences in size, centrality, and area covered were assessed. Advanced AMD was defined as presence of either CNV or GA. CNV was confirmed on angiography and GA was defined as an area of hypopigmentation >175 µm with a choroidal vessel in its base on colour photography. Fundus autofluorescence and Optical Coherence Tomography images were not available when this study was conducted. Any discrepancies in grading were resolved through adjudication by senior clinicians (LR, RG). Kappa for inter-grader and intra-grader agreement for the study graders ranged from 0.64 to 0.76 and from 0.60 to 1.00, respectively and has been published elsewhere.[25]

### Outcome Measures

Primary outcome was progression of non-advanced AMD to either advanced AMD or higher severity scores of non-advanced AMD. The safety of the use of simvastatin in people whose lipid profile did not warrant intervention with a lipid lowering agent was assessed by analysis of adverse events.

### Assessment of AMD progression

Progression was determined by comparison of AMD severity based on detailed AMD grading and confirmed by a masked side-by-side comparison of the baseline and the last follow-up images. Cases of disparity were reviewed with additional information from clinical examination and adjudicated where necessary.

AMD severity in each eye at baseline and at follow-up visits was assessed using a previously published [26], [27] 6-level severity scale based upon fundus features within a 6000 µm circle centred on the fovea, with higher levels indicating more severe disease. The severity scale was: Level 1 - hard drusen (<63 µm) only; Level 2 – intermediate drusen (64–125 µm) or hyperpigmentation only; Level 3 – large (>125 µm) soft drusen, without pigment change or intermediate drusen with pigment change; Level 4 – large soft drusen AND pigment change; Level 5 – GA within 3000 µm of the fovea; Level 6 – CNV. All participants with bilateral non-advanced AMD had a severity Level of 3 or above in both eyes at baseline, correspondent to ‘intermediate AMD’ in the Beckman classification of AMD.[21] Change of AMD status to a more severe level on this scale was considered as progression. Where one eye progressed to GA and the other eye progressed to CNV, we classified the participant as ‘progressed to CNV’ in ‘by person’ analysis (one case in each group).

To allow for smaller increments in AMD status to be considered as progression, those cases where there was an increase of 2 or more steps within the specific levels were also considered to have progressed. To assess this change we considered an increase in size, total number, area occupied by a lesion or movement to a more central location, as the within-level progression. Individuals who had a one step worsening in at least 2 characteristics were also classified as progressed (Table 1). Regression of early AMD features was also recorded.

**Table 1:** Macular characteristics used to determine severity in non-advanced age-related macular degeneration.

Macular Features	Maximal size (µm)	Number*	Most central location (distance from the fovea in µm)	Area affected in each location (as per column 4)
Intermediate drusen*	= 63<125	0	Further than 3000	0
Soft distinct drusen	= 125<250	1 to 9	1500 to 3000	<10%
Soft indistinct drusen	= 250	10 to 19	500 to 1500	<20%
Hyperpigmentation		20 or more	<500	<50%
Hypopigmentation			Foveal	>50%

*Category ‘Number’ is related to drusen only.

Masked side-by-side comparisons of baseline and 36 months visit images were performed independently for the whole sample by four graders, so that each eye was determined to be either the same, better, or worse in severity at follow-up when compared to baseline. If there was any doubt as to whether change has occurred, the images were scored as ‘same’. The side-by-side results were then matched with the results from the detailed grading of macular characteristics and discrepancies were resolved by consensus using all available clinical information. The side-by-side comparison allowed for a ‘whole picture’ approach in identifying small changes in AMD status that might not have been detected otherwise.[28]

### Genetic analysis

Genomic DNA was isolated from venous blood leukocytes using a standard phenol/chloroform extraction procedure. *APOE* genotyping was performed by multiplex high-resolution amplicon melting (TrendBio Pty Ltd, Melbourne, Australia).[29] Two primer pairs were designed to encompass 2 sites at amino acid positions 112 (site A) and 158 (site B) of the *APOE* gene. A sequence variant of c.526C>T for *???2* allele is present at site A (GenBank reference sequence NM_000041.2) or c.388T>C for *???4* allele is present at site B (reference sequence NM_000041.2) resulting in either a cysteine or arginine residue respectively. *CFH* genotyping for rs1061170 *(Y402H)* and rs2274700 SNPs was performed using the MassARRAY® platform (SEQUENOM) as previously described.[30]

### Statistical Analysis

Primary analysis was done on *intent to treat* basis and utilized logistic regression analysis to assess the effect of simvastatin on AMD progression, after adjusting for pre-specified co-variables of age, sex, smoking status, and also status of disease in the fellow eye (intermediate or advanced). Analysis was done ‘by person’ and used the data from the eye showing greatest progression. If one eye of a person worsened and the other eye showed improvement, the person was classified as having progressed. The latest available observation from those participants who finished the study earlier than 36 months was carried forward.

Secondary analyses included *on protocol*, *cross-over* (actual simvastatin use), and genetic analyses. In *cross-over* analysis, the participants who started on placebo and were then commenced on simvastatin by their general practitioner were analysed as being in the active treatment group. Additionally, as two treatment arms, despite the randomization, were uneven in proportion of participants with advanced disease in one eye (higher in the simvastatin group), we performed a *post hoc* analysis stratified by AMD severity in the fellow eye.

To address our second aim, we pre-planned to determine the modifying effect of apolipoprotein E (*ApoE*) gene single nucleotide polymorphisms (SNPS) on treatment efficacy, as the impetus for this study on simvastatin was based on our previous research that implicated involvement of the ApoE gene (a cholesterol pathway gene) in AMD development.[31], [32] Additionally, given the evidence for the association of AMD and its progression with complement factor H (*CFH*) gene, an exploration of the moderating effect of different genetic variants of the *CFH* gene on simvastatin treatment was also included in the statistical analysis plan.

The possible moderating influence of genotype on the effect of simvastatin was assessed through the tests of multiplicative interactions between treatment type (simvastatin versus placebo) and the *at risk* genotypes. Interactive effects were tested using a 2-stage sequential logistic regression model, with treatment type and genotype entered into the model at stage 1 and interaction between these 2 variables added in stage 2. Where statistically significant interaction suggested a moderating influence of genotype on the effect of simvastatin, we conducted further analysis of treatment outcome in placebo and simvastatin groups, stratified by genotype.

Adverse events and compliance with the assigned treatment of simvastatin and placebo were assessed using χ^2^ tests. Lipid profiles were compared between baseline and latest available follow-up measurement within a 36 months period using paired-samples t-tests, and differences in total cholesterol, HDL-C, LDL-C, and triglyceride levels between the two treatment groups at the end of follow-up were assessed using t-tests for independent samples.

### Sample size and study power

The natural history of AMD is that its severity in non-advanced features increases gradually over many years, ultimately progressing to sight-threatening advanced AMD. Phase 3 trials require many thousands of participants to be studied over many years to determine efficacy in reducing the risk of progression to advanced AMD [33], [34] This proof of concept study aimed to determine, with smaller numbers, if there was any efficacy signal in smaller degrees of progression so that we were interested not only in progression to advanced AMD but also in progression within the earlier stages of disease. Therefore, we calculated the sample size based on the previously observed rates of progression that included both the progression to advanced AMD and the estimates of the gradual increase in non-advanced AMD severity.[21]

The participants enrolled in the study presented a high risk of progression due to having either bilateral drusen >125 µm with or without pigmentary change, or multiple intermediate drusen and pigmentary change (12% to 50% five-year risk of progression to advanced AMD) or unilateral advanced AMD in one eye and any non-advanced AMD features in the other eye (35% to 53% five-year risk of progression to advanced AMD in the second progressing eye).[35] In addition, we also took as progression an increase in severity within non-advanced disease. For example, the risk of bilateral medium sized drusen (63 to 125 µm) becoming large drusen has been recently identified and reported as 40% in 3 years (Figure 5 from Ferris et al, 2013).[21] Given that our criteria for progression included small stepped increases in severity within non-advanced stages of disease, such as increases in size, number, area and centrality of drusen, we estimated that 50% of the study cohort will progress over 3 years according to the criteria outlined in this and other papers. [26], [27], [36]

To detect a 50% reduction in progression of disease (from 50% to 25%), with power of 80% and alpha = 0.05, we needed to study 58 subjects in each arm. Sample size calculations were performed with the PS - Power and Sample Size Calculation software.[37] The data were analysed using SPSS-18 statistical package for Windows (PASW Statistic 18, SPSS Inc, Chicago, USA). The Forest plot was constructed using StatsDirect statistical software version 2.7.9 (9/07/2012, http://www.statsdirect.com/), (StatsDirect Ltd, Altrincham, UK).

## Results

### Baseline characteristics

**Figure 1:**
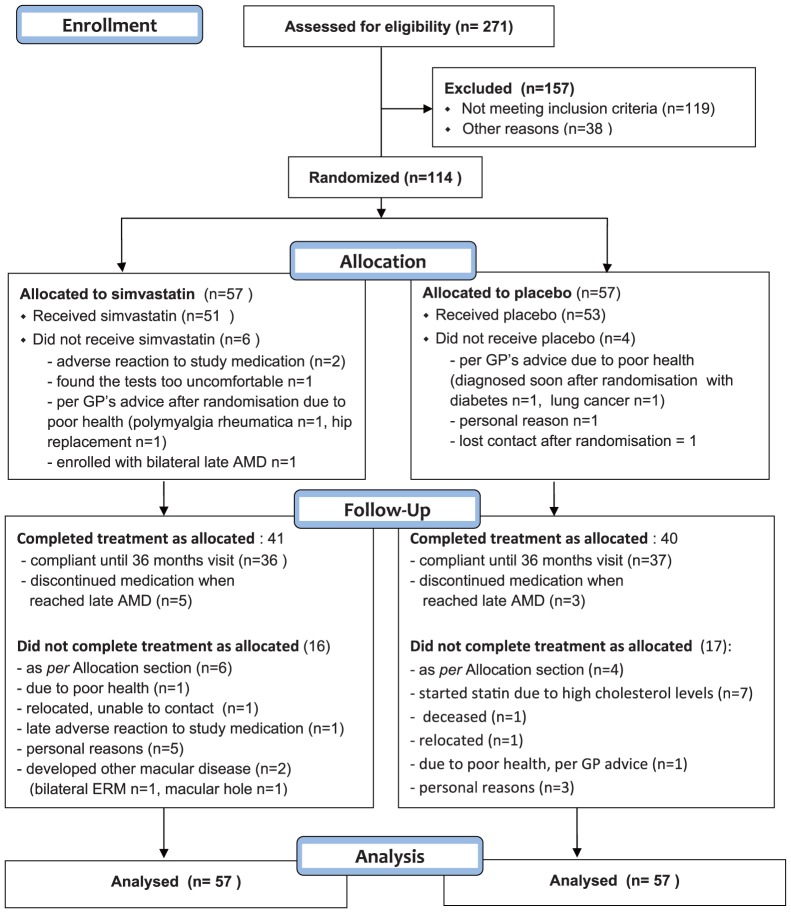
Flowchart of study participation.

A total of 114 participants were enrolled and randomized in 2003-2006 and followed up for three years, with 57 randomized to placebo and 57 randomized to active medication (Figure 1). Mean age of participants was 74.6±7.0 years; 77 (68%) were female and 60 (53%) were current or former smokers; 48 (42%) participants had advanced AMD, either GA or CNV, in one eye at baseline.

Baseline characteristics were similar between the two study groups, except that the number of participants with unilateral advanced AMD was twice as large in the simvastatin group compared to the placebo group (χ^2^_df = 1_ = 9.2, p = 0.002). Smoking was also less prevalent in the placebo group; the difference was marginally significant (χ^2^_df = 1_ = 3.5, p = 0.06) (Table 2).

**Table 2:** Baseline characteristics of placebo and simvastatin study groups.

Participant characteristics	Placebo n = 57	Simvastatin n = 57
Age, mean (SD), years	74.4 (6.4)	74.8 (7.5)
Women, No. (%)	38 (66.7)	39 (68.4)
Ever smoked, No. (%)	25 (43.9)	35 (61.4)
Advanced AMD in one eye, No. (%)	16 (28.1)	32 (56.1)
Supplements intake, No. (%)	38 (66.7)	33 (57.9)
History of cardiovascular disease, No. (%)	11 (19.3)	5 (8.8)
History of hypertension, No. (%)	23 (40.4)	18 (31.6)
Total cholesterol level, mean (SD), mmol/L	5.71 (0.78)	5.63 (1.06)
HDL Cholesterol level, mean (SD), mmol/L	1.86 (0.45)	1.78 (0.44)
LDL Cholesterol level, mean (SD), mmol/L	3.34 (0.66)	3.27 (0.97)
Triglycerides level, mean (SD), mmol/L	1.10 (0.39)	1.25 (0.51)
*ApoE* genotype, No. (%)		
* ???2/???3*	10 (18.9)	12 (23.1)
* ???2/???4*	3 (5.7)	2 (3.8)
* ???3/???3*	33 (62.3)	33 (63.5)
* ???3/???4*	7 (13.2)	5 (9.6)
*CFH* rs1061170 genotype, No. (%)		
* CC*	23 (42.6)	22 (41.5)
* CT*	24 (44.4)	27 (50.9)
* TT*	7 (13.0)	4 (7.5)
*CFH* rs2274700 genotype, No. (%)		
* CC*	30 (57.7)	36 (69.2)
* CT*	22 (42.3)	15 (28.8)
* TT*	0 (0.0)	1 (1.9)

### Association between AMD progression and simvastatin – total sample

At 3 years follow-up, the total progression of AMD from baseline was 31/57 (54%) individuals in the simvastatin group and 40/57 (70%) individuals in the placebo group (Table 2). This was mainly explained by the increased number of participants worsening in the severity of non-advanced AMD in the placebo group compared to the simvastatin group (49% vs. 32%, respectively, Table 3). When progression to advanced AMD was assessed, there were equal proportions of participants in both treatment arms: 12/57 (21%) in the simvastatin group (7 to GA and 5 to CNV) and 12/57 (21%) in the placebo group (9 to GA and 3 to CNV).

**Table 3:** AMD progression by treatment group.

	Placebo	Simvastatin
**At risk of progression by person,** No.	**57**	**57**
**Progressed total,** No. (%)	**40 (70.2)**	**31** (**54.4**)
Progressed to advanced AMD**,** No. (%)	12 (21.1)	12 (21.1)
Progressed, but not to advanced AMD**,** No. (%)	28 (49.1)	18 (31.6)
**At risk of progression by eye, No.**	**97**	**82**
**Progressed total,** No. (%)	**58 (59.8)**	**40** (**48.8**)
**Progressed to advanced AMD,** No. (%)	**16 (16.5)**	**14** (**17.1**)
Progressed to non-central GA**,** No. (%)	7 (7.2)	5 (6.1)
Progressed to central GA**,** No. (%)	6 (6.2)	4 (4.9)
Progressed to CNV**,** No. (%)	3 (3.1)	5 (6.1)
**Progressed, but not to advanced AMD,** No. (%)	**42 (43.3%)**	**26** (**31.7**)

**Figure 2:**
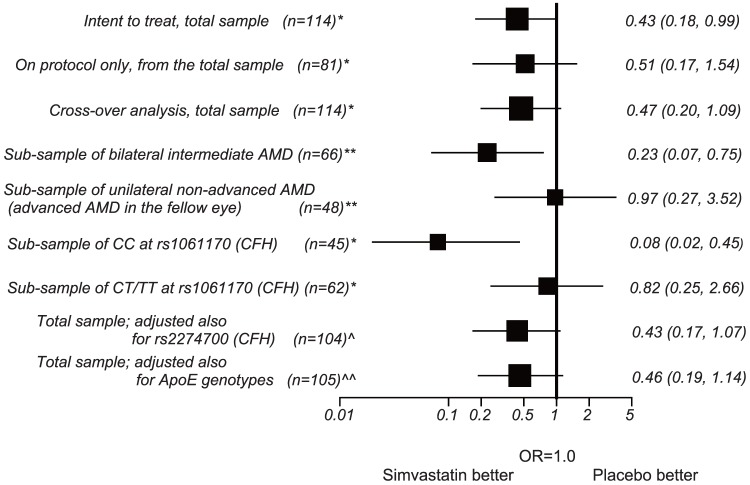
Forest plot of odds ratios (95% confidence intervals) for the effect of simvastatin on AMD progression from different models of the analysis.

The *intent to treat* univariate logistic regression analysis showed a tendency towards reduction of the odds of all AMD progression in the simvastatin group, although not statistically significant, with OR 0.51 (95% CI 0.23, 1.09), p = 0.08. In multivariate analysis, there was a significant reduction in AMD progression in the simvastatin group compared to the placebo group (OR = 0.43 (95% CI 0.18, 0.99), p = 0.047), after adjusting for age, sex, smoking, and unilateral advanced AMD status at baseline (Table 4 and Figure 2). Similar results were obtained in the *cross-over* analysis (adjusted OR = 0.47 (95% CI 0.20, 1.09), p = 0.08). In *on protocol* analysis, the effect of simvastatin was in the same direction although less significant (Figure 2).

**Table 4:** Logistic regression analysis of simvastatin effect on AMD progression.

Type of analysis	Unadjusted estimates			Adjusted estimates*		
	OR	95% CI	p-value	OR	95% CI	p-value
**Intent to treat, **total sample (n = 114)	0.51	0.23, 1.09	0.08	0.43	0.18, 0.99	0.047
**On protocol only,** total sample (n = 81)	0.78	0.29, 2.08	0.62	0.51	0.17, 1.54	0.23
**Actual use** of simvastatin (cross over), total sample (n = 114)	0.55	0.25, 1.20	0.13	0.47	0.20, 1.09	0.08
**Intent to treat, stratified by AMD status:**						
Subset of intermediate bilateral AMD (n = 66)	0.34	0.12, 0.96	0.04	0.23	0.07, 0.75	0.015
Subset of non-advanced AMD in one eye and advanced AMD in the fellow eye (n = 48)	0.88	0.26, 3.01	0.83	0.97	0.27, 3.52	0.96

*Adjusted for age, sex, smoking, and unilateral advanced AMD.

### Stratification by AMD severity at baseline (*post hoc* analysis)

*Intent to treat* multivariate logistic regression analysis, stratified by baseline severity (presence of unilateral advanced AMD), revealed no significant effect of simvastatin on AMD progression amongst those who already had advanced AMD in the fellow eye (OR = 0.97 (95%CI 0.27, 3.52) p = 0.96), after adjusting for age, sex, and smoking status. However, in the group with bilateral intermediate AMD at baseline, treatment with simvastatin resulted in a large reduction in the odds of progression compared to the placebo group (adjusted OR = 0.23 (95%CI 0.07, 0.75) p = 0.015) (Table 4).

### AMD progression by genotype and treatment allocation

Genotyping results were available from 105 participants for the *ApoE* gene. The majority of the participants (63%) carried the ???3/???3 genotype and 26% carried at least one *at risk* ???2 allele (Table 2); these frequencies are similar to the ones we have observed previously in a similar population.[38] In relation to the *CFH* gene, we conducted separate analyses for the two SNPs of the *CFH* gene known to be associated with the risk of AMD: rs1061170 (n = 107) and rs2274700 (n = 103). Very few individuals were homozygous for the T allele at either SNP (Table 2) which mirrored our previous findings in early AMD [30], hence they were aggregated with the CT genotype for the analyses. There was no departure from Hardy-Weinberg equilibrium for *ApoE* or *CFH* genetic variants (p>0.05).

In the *intent to treat* analyses we found a significant, 2-fold reduction in the odds of AMD progression associated with simvastatin treatment when rs1061170 (Y402H) was included in the multivariate model, (Table 5) which also included age, sex, smoking and unilateral advanced AMD. There was an interaction between simvastatin treatment and the CC genotype at the Y402H SNP of the *CFH* gene (p = 0.04), therefore we stratified the analysis by the Y402H genotypes of the CFH gene (Table 5). Logistic regression analysis stratified by Y402H genotype showed a highly significant 12-fold reduction in AMD progression in the group assigned to simvastatin if they were homozygous for the *at risk* C allele at Y402H of the *CFH* gene [OR = 0.08 (95%CI 0.02, 0.45), p = 0.004], but not in the combined group of CT and TT genotypes (p = 0.74) (Table 5). *ApoE* genotype did not influence the effect of simvastatin on AMD progression (p = 0.86) (Table 5).

**Table 5:** AMD progression by treatment allocation and genotypes of the *CFH* and *APOE* genes.

	Unadjusted estimates			Adjusted estimates*		
	OR	95% CI	p-value	OR	95% CI	p-value
**rs1061170 (Y402H) of the ** ***CFH*** ** gene**						
Simvastatin	0.46	0.20, 1.03	0.06	**0.40**	**0.16, 0.97**	**0.04**
* CC* genotype of the rs1061170	1.09	0.48, 2.49	0.83	1.13	0.48, 2.66	0.78
Interaction term “*CC* rs1061170 by simvastatin”			**0.024**			**0.04**
**Stratification by rs1061170 (Y402H) genotype of the ** ***CFH*** ** gene**						
1. Effect of simvastatin in the subset of participants with *CC* genotype	**0.13**	**0.03, 0.55**	**0.01**	**0.08**	**0.02, 0.45**	**0.01**
2. Effect of simvastatin in the subset of participants with *CT* or *TT* genotype	1.00	0.35, 2.83	1.00	0.82	0.25, 2.66	0.74
**rs2274700 of the ** ***CFH*** ** gene**						
Simvastatin	0.49	0.21, 1.12	0.09	0.43	0.17, 1.07	0.07
* CC* genotype of the rs2274700	1.28	0.55, 3.02	0.57	1.23	0.50, 3.01	0.65
Interaction term “*CC* rs2274700 by simvastatin”			0.21			0.17
***ApoE*** ** genotype**						
Simvastatin	0.49	0.22, 1.12	0.09	0.46	0.19, 1.14	0.09
* ApoE* genotypes (at least one *???2* vs. no *???2*)	1.44	0.58, 3.60	0.43	1.41	0.53, 3.75	0.49
Interaction term “*???2-*containing *ApoE* genotypes by simvastatin”			0.88			0.86

Note: Interactions between genotype and simvastatin were tested in a sequential regression model, with treatment group and genotype entered in the first stage and interaction between these two variables entered in the second stage. Coefficients shown are from stage 1 model for the treatment group and genotype variables and from stage 2 for interactions.

*Adjusted for age, smoking, sex and unilateral advanced AMD.

The analyses presented here are also summarised in Figure 2. As can be seen, the overall trend is for the direction of the effect to consistently favour simvastatin.

### Compliance with the study medication

Overall, 86/114 (75%) individuals, equally distributed between the two groups, were estimated to have consumed over 75% of their allocated tablets. At the three year follow-up visit, 41 (72%) of the simvastatin group and 40 (70%) of the placebo group either remained on their assigned medication and participated in the biannual reviews or had ceased the study treatment because they had reached advanced AMD in both eyes. Seven (12%) participants from the placebo group commenced cholesterol lowering medications prescribed by their physician due to an abnormal lipid profile (Figure 1).

Nine participants did not attend any follow-up examinations (5 due to poor health, 3 for personal reasons, 1 from an adverse reaction to the drug in the simvastatin group, 1 case was enrolled incorrectly, having advanced AMD in both eyes, and in one additional case, epiretinal membranes developed in both eyes, which precluded accurate photo-assessment of AMD progression. For these 11 records, baseline data on AMD status were carried forward and used as the outcome in *intent to treat* analyses.

Indirectly, compliance was also assessed through comparison of lipid profiles at baseline and the latest follow-up within 36 months. This information was available for 113 participants: 57 from the placebo and 56 from the simvastatin group. There was a significant difference between the two groups in mean changes in the levels of total cholesterol, LDL-cholesterol, and triglycerides between baseline and the latest follow-up tests, with lowering of the lipid levels by 20% to 25% in the simvastatin group and no significant changes in the placebo group. Both groups had a lowering of HDL cholesterol levels, with no difference between the groups (Table 6).

**Table 6:** Lipid levels at baseline and follow-up by treatment allocation.

Test	Treatment	Baseline	Follow-up	P value*	P value**
Total Cholesterol, Mean (SD), mmol/L	Placebo	5.71 (0.78)	5.66 (0.80)	0.53	0.001
	Simvastatin	5.63 (1.06)	4.47 (0.85)	0.01	
HDL Cholesterol, Mean (SD), mmol/L	Placebo	1.86 (0.45)	1.75 (0.43)	0.01	0.56
	Simvastatin	1.78 (0.44)	1.70 (0.44)	0.02	
LDL Cholesterol, Mean (SD), mmol/L	Placebo	3.34 (0.66)	3.36 (0.66)	0.76	0.001
	Simvastatin	3.27 (0.97)	2.31 (0.69)	0.01	
Triglycerides, Mean (SD), mmol/L	Placebo	1.10 (0.39)	1.18 (0.56)	0.12	0.001
	Simvastatin	1.25 (0.51)	1.00 (0.37)	0.01	

*P value from paired samples t-test, comparing baseline and follow-up measurements in each treatment group.

**P value from independent samples t-test comparing the differences (baseline level minus follow-up level) between the two treatment groups.

### Adverse events

Administering simvastatin to a cohort that would not have warranted lipid-lowering medications as a result of their lipid profile is not well studied and required surveillance of harm. In this study, we used both liver function tests and passive surveillance of adverse events that the study participants had spontaneously reported during follow-up assessments. The information on specific symptoms of possible side effects of statins, such as muscle pain and weakness, rash, mild and temporary headache, was provided to the study participants, and the importance of reporting such symptoms was explained at the time of consenting to the study.

Overall, 64 people reported at least one adverse event within the 36 months of follow-up, 25/57 (44%) in the simvastatin group and 39/57 (68%) in the placebo group (χ^2^_df = 1_ p = 0.008). Major illnesses were reported by 7 individuals in the simvastatin group and 15 individuals in the placebo group, and there was 1 death in the placebo group. Muscle aches, a recognized side effect of statins, were reported in 7 participants: 2 on placebo and 5 on simvastatin. As a result, 4 withdrew from the study (1 placebo and 3 simvastatin), 1 (placebo) stopped taking the assigned tablets and continued in an *off protocol* mode and 2 participants (both simvastatin) continued with the randomized treatment, as the symptoms settled. Two participants (one in each treatment group) were diagnosed with acute hepatitis. Otherwise, none of the participants had abnormal liver function tests that necessitated stopping medication.

In total, there was an absence of evidence of harm from using simvastatin in the dose of 40 mg daily.

## Discussion

This study reports the results from the first longitudinal proof-of-concept double-masked randomized placebo-controlled trial exploring the effect of the HMG Co-A reductase inhibitor, simvastatin, on slowing the progression of AMD. Our results indicate that dose of 40 mg daily was well tolerated in people with normal lipid profiles and that simvastatin appears to have a role in slowing progression of bilateral intermediate AMD. In those who had already developed advanced AMD in their fellow eye, we did not detect a beneficial effect for the eye with non-advanced AMD. The effect of simvastatin was more pronounced in those who were homozygous for the *at risk* C allele of the Y402H SNP of the *CFH* gene.

Almost all participants in this study had at least one C allele at Y402H, which is consistent with many AMD studies, including our own.[30] The reference group consisted mainly of individuals who were heterozygous at this SNP. However, as specific targeting of genetically predisposed individuals was not a factor in initial recruitment, this should not be considered problematic. The detection of the benefit of simvastatin predominantly amongst those homozygous for the at-risk CC genotype of Y402H of the *CFH* gene suggests that in future studies, genotype should be taken into consideration when assessing the potential effect of statins in AMD. Previous studies examining the effect of statins in AMD may have missed a beneficial effect by not stratifying their analysis by genotype.

Statins, initially used as cholesterol-lowering agents,[39], [40] have a variety of well recognized modes of action, some of which may benefit individuals presenting with AMD. By binding to HMG-CoA reductase's active site, statins inhibit hepatic cholesterol biosynthesis and reduce serum levels of low density lipoprotein cholesterol (LDL-C). The non-lipid-related actions of statins include improvement of endothelial functions, decrease of LDL oxidation, as well as reduction of inflammation and angiogenesis – all potentially important factors in AMD pathogenesis.[1], [2]

Our finding of a significant slowing of AMD progression due to simvastatin treatment (OR 0.43 (95% CI 0.18, 0.99), p = 0.047) was similar to the results obtained from a meta-analysis of case-control and cohort studies, where the pooled relative risk (RR) of developing AMD for all studies was 0.74 (95% CI 0.55, 1.00).[8] The prospect that there may be a differential effectiveness of simvastatin according to genotype is of great interest. It is not the first time that statins have been shown to work differentially in different populations. Rosuvastatin reduced the risk of major cardiovascular events in the group with no cardiovascular risk factors, apart from an elevated CRP, but it was most effective in those who showed a decreased CRP post statin. [41], [42]. It is possible that the lack of stratification by genotype has led to inconsistent results in previous studies investigating the association between statins and AMD. [3]–[16]

Statins are the most prescribed and used drugs in Australia [43], [44] and other Western countries and their use has increased within the last 10 years. The recent suggestion that there has been a decrease in AMD incidence in later birth cohorts raises the possibility that the recent wide spread use of statins to lower cholesterol levels may have contributed to the decline in AMD incidence.[45]

Recruiting participants into this study was extremely challenging, as many potentially eligible individuals with AMD were already taking statins or had lipid profiles where lipid-lowering agents were recommended. Whilst our study provides some support for a potential role for statins in AMD, a larger RCT would be required to provide a definitive result. With criteria for recommending statin use having widened in recent years, it will be even more difficult to attempt a RCT of statin use in AMD. It would, however, be possible to search for corroborating evidence by returning to the large population-based studies on AMD and repeat analyses, stratifying by genetic risk and the presence of unilateral advanced AMD.

The strengths of this study include its prospective, randomized, double masked design, the high rate of compliance, detailed grading of the macular photographic images, side-by-side assessment of baseline and follow-up images and the availability of angiographic findings to confirm CNV. The associations of AMD progression with age, smoking, and *CFH* polymorphism in this study were all consistent with other studies, indicating the similarities of our study cohort to the broader AMD-affected population. The limitations of the study are its relatively small sample size, the relatively high attrition rate, and a slightly higher number of participants in the simvastatin group who had no follow-up data. The use of only a moderate dose of simvastatin, and only three years of follow-up may also have limited the magnitude of the observed effect.

The relatively small sample size did not allow us to fully assess the effects of simvastatin on the incidence of advanced AMD. A moderate dose of simvastatin (40 mg per day) was chosen to minimize the risk of adverse events in a cohort of patients with normal lipid profiles; however there is a possibility that the effect could have been greater with a higher dose of simvastatin. As AMD progresses slowly, a longer follow-up could have provided more information on long-term effectiveness of simvastatin use in AMD. The observational Blue Mountain Eye Study was unable to detect any association of statins with AMD progression at a 5 year follow-up, [11] but after 10-years they were able to show that statins appeared to be associated with slowing the development of soft drusen.[7]

Although randomization was used to reach comparability between study arms, this randomization resulted in an imbalance in the distribution of smoking and advanced AMD in one eye at baseline between the two treatment groups. This imbalance meant that those most likely to progress (smokers and the unilateral advanced disease) were over represented in the treatment group. Although theoretically this made it more difficult to show a beneficial effect of the intervention, a protective association was still found.

In all sub-analyses the effect consistently fell on the side of favouring simvastatin. This is re-assuring and makes the chance association less probable. However given the sample size, the results stratified by the genotypes should not be overstated and further research into the use of statins in AMD, particularly profiled by genotype, would be invaluable. Statins are drugs with a well-defined safety profile and are currently taken by millions of people worldwide. If truly beneficial in slowing progression of AMD, their implementation would be low risk and rapid. The results presented here strongly indicate the importance of further determining the potential role of statins in slowing progression of AMD towards vision loss.

## Supporting Information

Checklist S1 **CONSORT Checklist.**
(DOCX)

Protocol S1 **AGE-RELATED MACULOPATHY STATIN STUDY (ARMSS) PROTOCOL.**
(PDF)
