# Supplementary material for: Proof of Concept, Randomized, Placebo-Controlled Study of the Effect of Simvastatin on the Course of Age-Related Macular Degeneration
Source: PLoS One. 2013 Dec 31;8(12):e83759. doi: 10.1371/journal.pone.0083759 (PMC3877099; doi:10.1371/journal.pone.0083759)
Supplement: Protocol S1 — AGE-RELATED MACULOPATHY STATIN STUDY (ARMSS) PROTOCOL. (PDF) [file pone.0083759.s002.pdf]

<u>A</u>GE-<u>R</u>ELATED <u>M</u>ACULOPATHY <u>S</u>TATIN <u>S</u>TUDY (ARMSS)

PROTOCOL

TABLE OF CONTENT

1. INTRODUCTION 2

2. STUDY RATIONALE 7

3. PERSONNEL 7

4. STUDY OBJECTIVES 8

4.1 ENDPOINT 8

5. STUDY DESIGN 8

6. PARTICIPANT POPULATION 10

6.1 SAMPLE SIZE 10
6.2 INCLUSION CRITERIA 10
6.3 EXCLUSION CRITERIA:- 11

7. STUDY MEDICATION 12

7.1 RANDOMISATION OF STUDY TREATMENT. 12

8. STUDY CONDUCT 9

8.2 PATIENT RECRUITMENT 9
8.3 INFORMED CONSENT. 9
8.4 STUDY ASSESSMENTS 11
8.5 ALLOCATION OF RANDOMISED STUDY TREATMENT 12

9. STUDY PROCEDURES 13

9.1 VISUAL ACUITY TESTING 13
9.2 DILATION OF PUPILS 13
9.6 OPHTHALMIC EXAMINATION 13
9.7 PSYCHOPHYSICS EXAMINATION 14
9.7.1 ROD PSYCHOPHYSICS 14
9.7.2 CONE PSYCHOPHYSICS 15

10. BLOOD SAMPLE 17

10.1 Equipment required for taking and processing of blood sample. *17*

11. EVALUATION OF SAFETY AND ADVERSE EVENTS 18

11.1 GENERAL ADVERSE EVENTS RELATING TO STUDY ASSESSMENTS 18
11.2 ADVERSE EVENTS RELATING TO OPHTHALMIC ASSESSMENTS. 18
11.3 SAFETY AND ADVERSE EVENTS RELATING TO STUDY MEDICATION. 18
11.4 REPORTING AND DOCUMENTATION OF ADVERSE EVENTS. 19
11.5 ABNORMAL LABORATORY RESULTS. 19

12. CARDIOVASCULAR ASSESSMENT 19

13. PHOTOGRADING 20

TERMINOLOGY AND DEFINITION OF AGE-RELATED MACULAR DEGENERATION. 22

APPENDICES 24

APPENDIX 1: LETTER TO OPHTHALMOLOGISTS 24
APPENDIX 2: FLYER FOR PATIENTS 25
APPENDIX 3: LETTER TO GP – INITIAL RECRUITMENT 26
APPENDIX 4: PARTICIPANT CONSENT FORM 27
APPENDIX 5: PARTICIPANT INFORMATION 29
APPENDIX 6: ADVERSE EVENT FORM 32
APPENDIX 7: ADVERSE EVENT FOLLOW-UP FORM 33
APPENDIX 8: LETTER TO PARTICIPANT ON COMMENCING MEDICATION 34
APPENDIX 9: LETTER TO GP ON COMMENCING MEDICATION 35
STUDY EXPLANATION 0

1. INTRODUCTION

Age-related macular degeneration (AMD) is a progressive, late onset disease affecting central vision that is currently the leading cause of irreversible blindness in our community. Early signs of the disease are present in 15% of the population aged 50 years and older and by 90 years of age, more than two-thirds of the population are affected[1]. The high prevalence of AMD, the anticipated increase in the ageing population and the very limited treatment options available highlight the urgency for further research and the implementation of a preventative strategy to retard the progression of this debilitating disease.

The pathogenesis of AMD remains largely unknown, however it is regarded as a genetic disorder where environmental risk factors impact on the genetic background. The potential environmental risk factors are numerous however, smoking[2] is currently the only factor consistently associated with AMD. Other cardiovascular risk factors such as hypertension, atherosclerosis, high serum cholesterol and high dietary fat intake have also been linked to AMD although inconsistently[3-6].

Recently, several pieces of evidence have further implicated cholesterol in AMD pathogenesis. Firstly, came the report that the cholesterol pathway gene apolipoprotein E (APOE) may play a role in AMD[7] and other degenerative diseases such as atherosclerosis[8] and Alzheimer's disease[9]. Secondly, the association of AMD with cardiovascular disease was reported in several studies.

Apolipoprotein E being a plasma lipoprotein plays a basic role in the degradation of particles rich in cholesterol and triglycerides. The polymorphic nature of the gene affects various diseases in different ways depending on which allele of the gene is present. That is, in cardiovascular disease the ε2 allele is protective and the ε4 allele a risk factor for disease[10]. In AMD, the ε4 allele has been shown to be protective whilst the ε2 allele was significantly associated with an increased risk of AMD[11].

There are many postulated mechanisms by which atherosclerosis could play a part in the development of AMD. A proposed model for the development of AMD is that deficiencies in the choroidal vasculature leads to deterioration in the retinal pigment epithelium resulting from the build up of waste products[12]. Blood flow to the choroidal vessels could be affected as well as the surface area of the choriocapillaris. The same mechanisms that cause lipids to be deposited in the walls of systemic arteries in atherosclerosis may also be involved in the deposition of lipids in crucial structures in the retina associated with AMD.

An overlap between the risk factors for cardiovascular disease and AMD have lead to the proposal that the two conditions may have a common aetiologic pathway. Components of cardiovascular disease such as hypertension and atherosclerosis have been studied in relation to AMD with inconsistent findings.

There have been several studies that have reported a significant association between hypertension and AMD[13-15]. Of particular interest are the findings from the AREDS group that reported an increased risk of large soft drusen or neovascular AMD in people with

hypertension[15].

The Rotterdam Study was the first population-based study to report a significant association between carotid atheroscleorsis determined by the presence of atherosclerotic plaques and late AMD[6]. This was supported by the findings of *Chaine et al* in 1998 who reported a significant association between coronary heart disease and advanced forms of AMD[16].

Dietary fat intake may also influence the risk of developing age-related macular degeneration by raising the levels of cholesterol in the bloodstream thereby increasing the risk of atherosclerosis or alternatively, there may be an increase in the deposition of fat in Bruch's membrane that adversely affects the flow supply of nutrients and the removal of waste products from the retinal pigment epithelium[17]. In recent years both the Blue Mountains Eye Study[18] and the Beaver Dam Eye Study[19] have shown that higher intake of specific fats associated with higher cholesterol levels were significantly associated with the presence of AMD.

Data collected from our population-based study of eye diseases (Melbourne Visual Impairment Project) was used to examine whether the progression of early AMD was delayed in people taking cholesterol-lowering medications. Our results showed for the first time, that participants on cholesterol-lowering medications were almost four times less likely to experience progression of AMD than participants who did not, although the major limitation was the small number of people who actually took such medications[20].

Further evidence to support our findings emerged from a survey of 379 men and women

residing in Sheffield, England. The survey found that men and women aged 66-75 who took statins had an eleventh the risk of AMD compared to those not taking the drug[21].

Given these findings, our aim is to assess the effect of a specific class of cholesterol-lowering medication ‘statins’ in reducing the progression of AMD in people with early signs of the disease. The rates of progression will be assessed in terms of APOE genotype to see if any correlation can be made.

People participating in this study will have fasting blood cholesterol levels below which are normally recommended to treat.

1. VanNewkirk M.et al Ophthalmology 2000; 107: 1593-1600
2. Smith W. et al Arch Ophthalmology 1996; 114: 1518-1523
3. Snow KK. et al Ophthalmic Epidemiol 1999; 6: 125-143
4. Klein R. et al Ophthalmology 1995; 142: 404-409
5. Smith W. et al Arch. Opthalmol. 1998; 116: 583-587
6. Vingerling J. et al Am. J. Epidemiol 1995: 142: 404-409
7. Souied E. et al Am. J. Ophthalmol 1998; 125: 353-359
8. Davignon J. et al Clin Chima Acta 1999; 286: 115-143
9. Strittmatter W. et al Proc. Natl. Acad. Sci USA 1995; 92: 4725-4727
10. Horejsi B et al Physiology Research 2000; 49: S63-S69
11. Klaver C. et al Invest. Ophthalmol &Vis. Sci 2001; 42: S310
12. Friedman E, et al Ophthalmology 1995; 102: 640-646.
13. Sperduto RD and Hiller R Arch. Ophthalmol 1986; 104: 216-219
14. Hyman L et al Arch. Ophthalmol 2000; 118: 351-358

15. AREDS group	Ophthalmology	2000; 107: 2224-2232
16. Chaine G. et al	BJO	1998; 8 : 996-1002
17. Mares-Perlman JA et al	Arch. Ophthalmol.	1995; 113: 743-748
18. Smith W et al	Arch. Ophthalmol.	2000; 118: 401-404
19. Seddon JM et al	Arch. Ophthalmol.	2001; 119: 1191-1199
20. McCarty C et al	MJA (letter)	2001; 175: 340
21. Hall NF et al	BMJ	2001; 323: 375-376

2. STUDY RATIONALE

Age-related macular degeneration (AMD) is currently the leading cause of irreversible visual impairment in our community. Despite numerous large, multi-centred studies aimed at finding treatments or prevention for AMD, the rates of blindness associated with the disease have not significantly decreased. New treatments mostly aimed at reducing visual loss once complications have occurred have had very little impact on this disease. It is therefore likely that prevention or slowing of the progression will have an important impact on this disease until better treatment is available.

Our recent findings within the department have shown that the use of cholesterol-lowering drugs may offer the first proven intervention available for slowing the progression of AMD to the late stages where severe vision loss is experienced. We therefore commence in 2003 a randomised-controlled trial of cholesterol-lowering agents, 'statins' in people with early AMD. The APOE gene allele status of each normocholesterolaemic individual will be determined to investigate whether the APOE allele type influences who will and will not progress on this medication.

3. PERSONNEL

Chief Investigator:

Dr. Robyn Guymer *(Centre for Eye Research Australia, University of Melbourne)*

Research Fellow:

Dr Luba Robman *(Centre for Eye Research Australia, University of Melbourne)*

Research Assistants:

Ms Mary Varsamidis *(Centre for Eye Research Australia)*

Mr Peter Dimitrov *(Centre for Eye Research Australia, University of Melbourne)*

Dr Khin Zaw Aung *(Centre for Eye Research Australia)*

Ms Nicole Hunt *(Centre for Eye Research Australia)*

Ms Theresa Dolphin*(Centre for Eye Research Australia)*

Pharmacist: Mr Hamish McCracken *(Royal Victorian Eye and Ear Hospital)*

Safety Officer: Dr Helena Teede *(Monash University, Department of Medicine and Vascular Science)*

4. STUDY OBJECTIVES

The primary objective of this study is to determine whether SIMVASTATIN at 40mg per day versus a placebo affects the progression of early AMD, either to the development of late stage AMD, defined as either CNV or central geographic atrophy, or as worsening macular status within the stage of early AMD.

Secondary objectives are to measure changes in visual function results over time and to determine how the genotype influences the findings on AMD progression in normocholesterolaemic participants.

4.1 Endpoint

The endpoint of the study is the development of late stages AMD. If late AMD develops in both eyes, the participant exits the study and no further scheduled examinations will be required. If late AMD occurs in one eye, the fellow eye remains under observation and the follow up continues.

5. STUDY DESIGN

The study is a randomised, double-masked, controlled study. The study is based at the Royal Victorian Eye and Ear Hospital and Caulfield General Medical Centre. .

Participants are being randomised in a ratio 1:1, to receive either the active ‘statin’ or the placebo once they have completed both the ophthalmic and cardiovascular requirements outlined in the protocol.

Participants receive the same study treatment for the duration of their involvement in the study.

6. PARTICIPANT POPULATION

6.1 Sample size

In photo-based detection, the rate of progression of early AMD in people with high- risk features was found to be 5% per year in the AREDS study[22]. In the VIP project, AMD progression in people on cholesterol-lowering medications was 4 times lower than those not on any such medication[20]. To detect a similar reduction in AMD progression, a sample size of 118 subjects in each arm, followed for three years, was needed to reach 80% power at a 5% level of significance.

20. McCarty et al	MJA	2001 175: 340
22. AREDS	Arch Ophthalmol	2001 119: 1417-1436

In the more sensitive functional tests, a smaller sample size would be required. Thus, a sample size of 50 patients per treatment group would be sufficient to detect a 22% to 41% change in sensitivity for the different functional methods, or it will be sufficient to detect a 3.4 min difference in rod-cone break over the trial period, with statistical power of 80% .

6.2 Inclusion criteria

(1) male or female aged 50 years or older,

(2) the ability to assess the macula in at least one eye,

(3) VA better than or equal to 6/18 in the study eye(s).

(4) High risk drusen in both eyes – ≥1 large soft druse (125 microns), or >10 intermediate drusen (62.5 microns)

OR

Late AMD (CNV, GA) in one eye and any drusen or pigment change in the

study eye.

Note: the study eye is allowed to have non-central GA and/or non-neovascular PED

(5) cholesterol level within the normal limits according to the person's medical history

(6) not currently on any cholesterol-lowering medication.

6.3 Exclusion criteria:-

(1) Medical and ophthalmic conditions which potentially affects visual function – including visually significant cataract (as defined by WILMER's grading method-that is nuclear opacity score of 2.00 or greater, cortical opacity score of greater than 3, any posterior subcapsular cataract), history of diabetes and glaucoma

(2) Use of medications which may affect visual function, such as plaquenil, chloroquine, major tranquilizers.

(3) cholesterol levels outside the normal limits given person's medical history.

- Existing coronary heart disease:-- **cholesterol > 4 mmol/L**
- Diabetics, people with hypertension, peripheral vascular disease, family history of hypercholesterolaemia or coronary heart disease:-- **cholesterol > 6.5mmol/L or cholesterol > 5.5 if HDL <1.0mmol/L**
- people with HDL <1.0mmol/L:--**cholesterol >6.5mmol/L**
- men aged 35 to 75 years or postmenopausal women up to 75 years:-- **cholesterol >7.5 or triglycerides > 4.0mmol/L**
- people with none of the above:--**cholesterol >9.0 or triglycerides >8.0mmol/L**

(4) Statins is clinically contraindicated

- Allergy
- ALT > 2 times the upper limit of normal
- Previous severe adverse reactions to statin

7. STUDY MEDICATIONS

Dose: The medication involved in this study are either SIMVASTATIN at 40mg per tablet or a PLACEBO.
Both are supplied by Merck Sharp & Dolme Pty Ltd.

Administration: Patients receive their study medication following the completion of their baseline assessment.

Unused tablets and empty jars are requested to be returned to the study centre for assessment of compliance.

7.1 Randomisation of study treatment.

Randomisation of study medication has been computer generated with 50% of participants being randomised to receive the placebo and 50% the ‘statin’. Staff involved in the examination of participants are masked as to who receives the placebo or the active ‘statin’.

8. STUDY CONDUCT

8.2 Patient recruitment

Participants are being recruited from both public and private ophthalmic clinics.

Letters have been sent to all Victorian ophthalmologists (Appendix 1). The letter has outlined the aims and requirements of our research study and also contained a flyer (Appendix 2) that can be given to those patients who express an interest in participating. The flyer provided a brief outline of the study and what is involved for the participants as well as providing a contact number for the study centre. Also included may be a letter that patients can take to their GP explaining the need for a cholesterol test (Appendix 3).

Potentially suitable subjects are contacted by research staff, who outline the objectives of the study, the requirements for the participant and order a set of baseline blood tests (including fasting lipids, LFT, CRP and hsCRP). Questions are asked regarding cardiovacular disease and risk factors status, in order to interpret the test results. The participant are notified of the blood test result regardless of whether it is normal, including suggestion of further actions (such as seeing his/her GP). If the blood test result is within the acceptable range for recruitment in the study then further details of the study is discussed with the participant, and prerandomization/baseline visits are arranged.

8.3 Informed consent.

Upon arrival at the study centre, the purpose, aims and requirements of the study are being explained to the participant by a Study Investigator or Research Assistant. Participants are being given the Patient Information Sheet (Appendix 3) and have an opportunity to ask any questions before they are asked to sign the informed consent form (Appendix 4). This forms points out that the participant has received an explanation regarding the aims of the study that the requirements of the study have been outlined to them, that their participation is voluntary and they consent to the ophthalmic and cardiovascular examinations and to the taking of a blood sample by a trained staff member for cholesterol, liver function testing and for DNA analyses.

(A separate consent form needs to be signed specifically for the DNA analyses.)

A separate agreement to our privacy policy has to be signed.

9

8.4 Study assessments

The assessments required for the study include:

Questionnaire

(1) Ethnic origins and family history

(2) History of cardiovascular disease and risk factors

(3) Medications – presciption and over the counter

(4) Functional questionaire (IVI, or Impact of Visual Impairment Questionaire)

(5) Dietary questionaire

(6) Cognitive function assessment—*Mini-mental questionnaire*

ii) Ophthalmic examination

(1) Refraction and vision testing

(2) Pupil dilation

(3) Examination of retina

(4) Macular photographs

iii) Cardiovascular examination

(1) blood pressure measurements.

iv) Blood sample- *overnight fasting blood samples are being taken to measure-*

(1) fasting lipid profile, including total cholesterol level, HDL- and LDL- cholesterol, triglyceride level, liver function test, CRP and hsCRP

(2) and for DNA analyses *(this blood is to be sent to the McComas Laboratory as part of the ongoing AMD inheritance study [AMDIS: approval number:95/283H/00] examining potential genes involved with AMD. At present the genes of great interest are those involved in the cholesterol pathway such as the APOE gene).*

v) Psychophysics testing, which measures the subtle functions of the eye.

(1) Rod component – recovery time constant and absolute threshold

(2) Cone component – recovery time constant and absolute threshold

(3) The rod cone break

8.5 Allocation of randomised study treatment

Once participants have completed all baseline assessments they receive their either simvastatin 40mg or placebo. For tracking, each participant is allocated a study medication number which is to be recorded in the database as well as a log book. Each medication number will correspond to either active medication or placebo, which is unknown to the participant and the study team.   Participants will keep the same medication number for the duration of their participation in the study.

9. STUDY PROCEDURES

Refraction and visual acuity will be performed for all participants by a trained vision examiner.

9.1 Visual acuity testing

Best-corrected visual acuity is measured using a retroilluminated ETDRS chart, using the EDTRS scoring system.

- Visual acuity is assessed with participant's most comfortable presenting correction
- (monofocal for distance, bifocals or multifocals) if applicable.
- Participant is seated 4 metres from the ETDRS visual acuity chart.
- Conventionally, the right eye is examined first, with the left eye being occluded.
- The participant is asked to select the line they can read most comfortably and is then encouraged to continue until they cannot read any further with accuracy.
- If fewer than 20 letters are read, the chart is moved to 1 metre.
- If the participant cannot read the top line at 1 metre, vision is checked with a pinhole.
- The distance and chart used are then recorded.
- Scoring of visual acuity is worked out according to the number of letters read correctly for each eye.
- Procedure is repeated for the left eye.

9.2 Dilation of pupils

One drop of tropicamide (0.5%) and phenylephrine hydrochloride (10%) in each eye is administered for pupillary dilation.

9.6 Ophthalmic examination

Ophthalmic examination is done with a standard slit lamp to assess the retinal changes related to AMD. It also allows assessment of any other abnormality of the ocular media and retina which may complicate

assessment. Retinal photography is performed with a standard fundus camera.

9.7 Psychophysics examination

Pupils are dilated using either one drop of tropicamide (0.5%) and phenylephrine hydrochloride (10%) in each eye, or one drop of oxybutacaine (0.5%) followed by one drop of tropicamide (1%). Psychophysics examination has 2 components:

1) Rod psychophysics
2) Cone psychophysics

9.7.1 Rod Psychophysics

Rod psychophysics is performed using the dark adaptometer. This utilizes a circular test target of white light, 2 degree size at 2 degree ecentricity. A small red fixation target is used. Initially there is a bleaching period of 5 minutes, after which all light is turned off. The participant is asked to fixate on the red target. The test target is made to appear at approximately 1 minute intervals. The participant is asked to indicate when he/she first sees the light, as soon as it appears, and as quickly as possible. The intensity of the light is then recorded.

The test is to be continued until the rod component has near "flattened out" in the last 2-3 readings, or if the rod-cone break has not been reached after 45 minutes of dark adaptation.

Total duration of test = 0.5 to 1 hour

9.7.2 Cone Psychophysics

Subjects are given practice at all tasks before beginning tests.

Subjects are given rests regularly and when required.

- Visual Acuity measurement: One eye is covered and the subject is asked to read the smallest line on a standard logMAR letter chart with the test eye (the subjects' current spectacles or best correction are worn.) (1 min)
- Ishihara test: Subjects are asked to read the numbers on the Ishihara test plates as a screening test for congenital colour vision deficiencies. (1 min)
- Spatiotemporal threshold test: Subjects respond in a yes/no manner to a series of targets presented on a computer screen. One eye is occluded with the use of trial spectacles. The targets viewed are centrally positioned, constant spatial frequency (4c/deg) targets. The temporal frequency is varied (1, 5, 10, 16Hz) and contrast sensitivity is found at each temporal frequency. This test may be repeated. (5-6 mins)
- Colour threshold test: Subjects respond in a yes/no manner to a series of targets presented on a computer screen. One eye is occluded with the use of trial spectacles. The targets viewed are centrally positioned, coloured blobs of red, blue or white. Contrast sensitivity is found for each colour. This test may be repeated. (3-4 mins)
- Visual Field tests: Subjects' 10° peripheral fields are tested with various stimuli: 1 - white; 2 - white flicker; 3 - red; 4 - red flicker. Subjects are asked to maintain central fixation and respond when peripheral lights are seen. One eye is occluded with an eye patch. (4-5 mins each, 20 mins total)
- Adaptation time: An estimate of subjects' sensitivity to a 5° spot, flickering at 5Hz, is made. One eye is occluded with the use of trial spectacles. Subjects look at a bright light for 40 seconds. Subjects then respond verbally when vision has recovered sufficiently to perceive the 5Hz spot at four times and two times the estimated threshold contrast. The time taken for this recovery is recorded. (1-3

mins)

- Blue-on-yellow visual field test: Subjects' 10° fields are tested with blue stimuli on a yellow background. Subjects are asked to maintain central fixation and respond when the peripheral blue lights are seen. One eye is occluded with an eye patch. (13-16 mins)

Total time: 1.5 – 2 hours

10. BLOOD SAMPLE

10.1 Equipment required for taking and processing of blood sample.

- Eye protection equipment e.g. goggles
- Latex gloves (optional)
- Tourniquet
- Alcohol swabs
- Cottonwool
- Micropore tape or Band-aids
- Ice if required for blood storage
- Freezer for storing blood
- Vacutainer needle
- Vacutainer barrel
- Vacutainer blood collecting tubes
- Vacutainer holder (“mushroom”)
- Sharps disposal bin
- Foam/Plastic tube holder
- Centrifuge

11. EVALUATION OF SAFETY AND ADVERSE EVENTS

11.1 General adverse events relating to study assessments

- Blood test will be performed by trained staff, but may cause minor discomfort and possibly a small bruise at the site.
- Cardiovascular measurements are all non-invasive and are not associated with any pain or discomfort.

11.2 Adverse events relating to ophthalmic assessments.

- Participants will experience blurred vision for several hours following the administering of the dilating drops; this is often associated with greater sensitivity to sunlight. It would be our recommendation for participants to bring their own sunglasses with them.

11.3 Safety and adverse events relating to study medication.

'Statins' in general, are reported to be well tolerated in the majority of individuals, with side-effects being mostly mild.

Simvastatin has been used in clinical studies at doses of up to 40mg with the reported side-effects including:

- skin rash (<4%),
- sore muscles (4%),
- transient and mild headache (4-8%),
- myalgia (2%),
- abnormal liver function levels and increased creatine kinase, aspartate aminotransferase and alanine aminotransferase levels.

11.4 Reporting and documentation of adverse events.

At each visit, research staff will question all participants regarding any adverse experiences particularly muscle soreness as an indicator of possible rhabdomyolysis. Any illness, sign, symptom or clinically significant laboratory test abnormality that appear or worsen during the course of the clinical trial will be documented and pursued regardless of the causal relation to the active 'statin' therapy. Participants would also be encouraged to report any problems as they occur to the study centre where all details would be documented and upon the Chief Investigator's discretion further action would be recommended to the participant.
(Adverse Event form located in Appendix 5).

Laboratory blood analyses and repeat liver function test carried out after 4 weeks will allow the monitoring of changes in cholesterol levels as well as liver enzymes.

The safety issue of the study would be evaluated by a Safety Officer appointed to review all the reported incidents of adverse events between the two groups.

11.5 Abnormal laboratory results.

Participants with laboratory results from the blood samples that appear outside the 'normal' range will be notified and a suitable course of action will be suggested such as the need to see their GP.

12. CARDIOVASCULAR ASSESSMENT

- blood pressure measurements.

13. PHOTOGRADING

Digital photography of the retina.

In this study, non-stereoscopic 45° macular photography using a digital Canon CR10-45NM Non-Mydriatic Retinal Camera with a resolution of 3072 x 2048 pixels will be used. Images will be viewed immediately and repeated if unsatisfactory. Images are then transferred onto a portable hard disk at the test site to allow them to be backed up on the central server at CERA each week, where additional image storage space has been purchased specifically for this project. Images will be graded using the "OptoMize PRO" software from Digital Healthcare Image Management System (UK). This software has measurement tools to estimate the size of the retinal lesions, and a grid covering macular area of 6,000 micron in diameter, to determine location and closeness of the lesions to the fovea. The grid is calibrated for each eye using the optic disc as a size reference.

Non-mydriatic fundus photography has been shown by Klein et al. to provide a reliable means for AMD research.(23) Since this publication, digital technology in retinal research has undergone further improvement, allowing retinal images to be taken through pupils of less than average diameter, as well as providing over 10 times greater image resolution. Direct comparison has now been shown that digital imaging technology to be as good as 35 mm film photography for the detection and grading of AMD features. (24)

AMD grading

Grading of macular characteristics uses the International Classification and Grading System for Age-related Maculopathy and Age-related Macular Degeneration. (25) Each digital photo is graded by two experienced graders for the absence or presence, as well as size, number, location, centrality and area covered by the following features associated with AMD: intermediate and soft drusen, retinal pigment epithelium changes, geographic atrophy and neovascular changes. The severity of AMD is assessed

according to the stepped increase in grading. Detailed grading will allow for analysis using any of several AMD definitions and grading schemes.

Detailed results from grading will be manually entered on a specifically designed grading sheet and then entered into the custom-written MS Access database.

The grading classification is based on the anatomical definition of the macula i.e. that part of the retina centred on the fovea in which the ganglion cell layer is more than one cell in thickness and that has an appropriate diameter of 5.5mm. For descriptive purposes, the inner macula is defined as the area within a circle centred on the foveola of diameter 3000 microns or approximately two disk diameters across. The outer macula is defined as the area between the inner macula (diameter 3000 microns) and a circle of 6000 microns.

The presence or absence of any feature in the macular area (within 6000 microns diameter across the macula centred on the fovea) was documented on a specifically designed grading sheet.

To enable the study to examine the role of vitamin E on early AMD features all drusen types within the grid area, their maximal size, total number, and area covered within each grading circle were documented and subsequently entered into a database. Other than these two additions to the AMD International group's guidelines, the definitions and protocol were strictly adhered to.

Trained graders are being involved in grading the study slides. The grading grid consists of three circles concentric with the centre of the macula and two perpendicular diagonal crosshairs. The diameter of the three circles referred to as the central, middle and outer areas correspond to 1000 microns, 3000 microns and 6000 microns respectively in the fundus of an average eye. The perpendicular diagonal cross hairs

subdivided the grid into subfields, which permits detailed grading of lesions in each subfield. The grader positions the central circle of the grid over the centre of the macula of the images. The measuring circles C_o, $C_{1,}$ $C_{2,}$ C_3 and $C_{4,}$ used to estimate size of drusen and the area involved by hypo- or hyperpigmentation, represent circles with diameters equivalent to 63 microns, 125 microns, 175 microns 250 microns and 500 microns respectively.

The absence or presence of all drusen types is recorded onto the grading sheet. For hyper- and/or hypopigmentation, the maximum size and the more central location were indicated. For cases of geographic atrophy and neovascular AMD features the maximum area it encompassed and the more central location was scored.

Reliability sessions will need to be carried out to assess intra- and inter- grader reproducibility. Graders remain masked to both the study treatment and cardiovascular health of all study participants. The reported agreement was based on the ability to correctly identify the presence or absence of all features thus an unweighted kappa statistic with 95% confidence interval was used as a measure of reliability.

Level of agreement represented by Kappa values are:
0.21-0.40 represents fair agreement,
0.41-0.61 represents moderate agreement,
0.61-0.80 represents substantial agreement and
0.81-1.00 represents almost perfect agreement.

Terminology and definition of Age-related Macular Degeneration.

The definition of AMD followed the ‘International AMD Epidemiological Study Group’s’ guidelines and

definitions. AMD is a disorder of the macular area of the retina characterised by a combination of the following primary features being present within a 6000 microns diameter of the fovea without indication that they are secondary to another disorder (e.g. high myopia):-

(a) soft intermediate drusen (drusen having fuzzy edges and decreasing density from the centre outwards ranging in size from > 63 microns to < 125 microns),

(b) soft distinct drusen (drusen having uniform density with sharp edges),

(c) soft indistinct drusen (drusen having decreasing density from the centre outwards with fuzzy edges),

(d) soft distinct and soft indistinct drusen,

(e) soft drusen associated with areas of increased pigment or hyperpigmentation and

(f) soft drusen associated with areas of depigmentation or hypopigmentation of the retinal pigment epithelium without any visibility of choroidal vessels

Drusen were categorised as:

Hard drusen: (all drusen < 63μm and drusen > 63μm but < 125μm with sharp edges).

Intermediate soft drusen: (> C_o ≤ C1; > 63μm ≤ 125μm with fuzzy edges)

Large, soft distinct drusen: (> C1, 125μm)

Large, soft indistinct drusen: (> C1, 125μm)

23. Klein R, Meuer S, Moss S et al. Ophthalmol 1992;99:1686-92.
24. van Leeuwen R, et al. Ophthalmol 2003;110:1540-4.
25. Bird A, et al. Survey Ophthalmol. 1995;39(5):367-74.

APPENDICES.

APPENDIX 1: Letter to Ophthalmologists

Dear xxx,

Re: ARMSS (A new trial of Cholesterol lowering medication in AMD)

As part of the ongoing research into Age related Macular Degeneration, we are commencing a new study to examine the possible role of cholesterol-lowering medication ('statins') in slowing the progression of AMD. The impetus of this comes from results of 'Melbourne Visual Impairment Project' that has indicated for the first time, that participants on cholesterol-lowering medications were almost four times less likely to experience progression of AMD than participants who did not (McCarty C et al MJA. 2001 volume 175, page 340).

This study is a collaborative project between Centre for Eye Research Australia and the Department of Optometry, The University of Melbourne. The study is a 3-year, randomised trial that would involve participants undergoing a fundus examination and a cardiovascular assessment. 50% of participants will receive the cholesterol-lowering medication and the other 50% will receive a placebo. Participants need to have normal lipid levels and cannot be on lipid lowering medication for this study.

We are therefore very keen to recruit potential participants with high risk fundus changes of AMD (see below for definition) and therefore seek your assistance in identifying suitable patients who may be interested in participating in either of our studies.

Enrolment criteria:

- **Age >50 years**
- **Patients need to have high risk AMD:**
- **Bilateral drusen: at least 1 large druse (≥ 125 microns) OR extensive intermediate drusen (63-125 microns) in both eyes , OR**
- **End stage AMD in one eye (GA or neovascularization) and any changes of AMD in the second eye**
- **visual acuity of 6/18 or better in the better eye**

We have included an information sheet for your patients and a letter they can take to their GP to arrange a fasting blood lipid test as this result will determine which study they could enter.

We hope that you will be able to copy these sheets for interested patients. Please contact Nicola Hunt, on 9929 8360 regarding particular patients so a suitable time can be arranged to see them in a study clinic, or write to us with the patients details and we will be very happy to contact them.

Many thanks for taking the time to assist us with our research into AMD.

Yours sincerely,

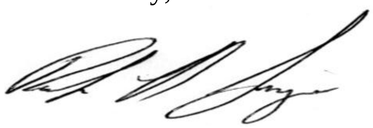

Robyn Guymer MB MS, PhD, FRACO,
Macular Degeneration Unit
Centre for Eye Research Australia. **Tel: 99298393, Mob: 0418618227**

APPENDIX 2: Flyer for the patients

Age-related maculopathy Statin Study (ARMSS)

Will the cholesterol lowering medication “statins” slow the progression of AMD?

Age-related macular degeneration (AMD) is the leading cause of legal blindness in the elderly population. You have some age-related changes in your eyes that put you at significant risk of progressing to the late complications of AMD resulting in loss of central vision in one or both eyes (one eye may already have this complication). At present there is no effective way to reduce your risk of this progression except for giving up smoking if you smoke. Otherwise eating a good diet of green leafy vegetables and possibly corn may help but we have no proven treatments to reduce the risk of progression of this disease.

We have some early results that suggest that cholesterol-lowering medication may have a role in reducing the risk of AMD progression. These are only very preliminary results and have proven nothing at this stage. However, we are hopeful that these medications might reduce the amount of fat in the back of the eye (irrespective of your blood cholesterol level) and thereby lower the risk of severe vision threatening complications. It is the accumulation of lipid or fatty deposits behind the retina that is thought to lead to bleeding or degeneration that occurs as a complication of AMD.

As a result of our preliminary study we commenced a 3 years study to test whether statins, a family of cholesterol lowering medication, can effectively slow the progression of early AMD. Over 900 people will be enrolled and half will take the active drug whilst the other half will be on a placebo (no active drug). Both drugs will look identical so neither yourself nor the investigating team will know which group you are in for the 3 years of the study.

You will be asked to do a fasting cholesterol test, and if it is within a certain normal range you would be a suitable candidate for this study. If your cholesterol level is high then you should discuss this with your doctor and you cannot be included in the study as you might be selected to the placebo group which would not be right if your cholesterol was at a level that needed attention. Regardless of your cholesterol level, you will be notified of the result and what action to take.

The medication for the study (Statins) has been used for many years to lower the risks of heart disease, and is generally very well tolerated. People in the study will take the medication for 3 years. Over this time, monitoring would involve examinations every 6 months. Each set of examinations will involve going to either Caulfield General Medical Centre in Caulfield, or Royal Victorian Eye and Ear Hospital in East Melbourne). The thorough examination ensures that we monitor not only the physical appearance in the eyes but also the visual function. The finer details of the study will be discussed with you should you become eligible, and agree to enter the study. In the meantime, if you have any questions please call Ms Nicola Hunt or Dr Robyn Guymer on 99298360.

Thank you once again for expressing interest in age-related maculopathy Statin Study.

Dr Robyn Guymer, MB BS, PhD, FRACO
Head of Macular Research Unit,
Centre for Eye Research Australia, University of Melbourne

APPENDIX 3: Letter to GP – initial recruitment

Dear General Practioner,

The Centre for Eye Research Australia (CERA) in collaboration with the Department of Optometry at the university of Melbourne are currently conducting a clinical trial examining the eye disease Age-related Macular Degeneration (AMD).

This study is a 3-year randomised controlled trial assessing the role of Simvastatin as a cholesterol-lowering medication, in the progression of early AMD. Participants in this study will receive an ophthalmic examination that includes photographs of the macular area. Participants enrolled in this study will be randomly assigned to the active or placebo arm of the trial. To take part in this study the participants must have lipids levels that are within a range that treatment is **not** recommended, as half will be on placebo.

Your patient has been identified by their ophthalmologist as having signs of AMD that fit our inclusion criteria and has shown interest in taking part in one of our research studies.
We need to know what the fasting blood lipid levels are to determine which study they can take part in. Therefore, we would appreciate if you could organise for your patient to have their fasting cholesterol level assessed. (We require: total cholesterol level, HDL and LDL- cholesterol and triglyceride level). Or provide them with a copy of a recent test (with in the last month) so that they can bring it to their initial study visit.

Should you require further information please feel free to contact me on
9929 8393 or 0418618227.

Many thanks for your time and assistance.

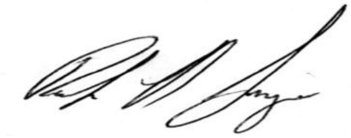

Robyn Guymer
Head of Age-related Macular Degeneration Unit
CERA.

APPENDIX 4: Participant consent form

<u>CONSENT FORM</u> FOR PARTICIPANTS WITH AMD IN THE CLINICAL STUDY:
"The Role of Cholesterol-lowering medications ('statins') in the progression of Age-related Macular Degeneration"

I……………………………………………………………………………….

hereby consent to participate in the clinical study evaluating the role of **statins** in the eye disease Age-related Macular degeneration.

I am aware that my participation in this study is voluntary and I am free to withdraw at any stage without this hindering any further medical treatment I may require.

I acknowledge that the aims, requirements, risks and side effects of this clinical trial as outlined on the "Patient Information" sheet, have been explained to me.
I hereby consent to having:

- my pupils dilated for the purpose of having my macula photographed
- undergoing non-invasive psychophysical testing (tests which subtly measure how well you can see)
- undergoing non-invasive cardiovascular assessment
- having a blood sample taken at each visit
- taking the study medication allocated to me.

I **DO/DO NOT** give permission for the study investigators to access my medical records should they require further information than I can provide regarding my medical history.

……………………………………………… ……………………
Signature of participant **Date**

………………………………………………. …………………….
Signature of Investigator /Research Assistant Date

APPENDIX 4a: Participant consent to continue the study form

<u>CONSENT FORM</u> FOR PARTICIPANTS WITH AMD IN THE CLINICAL STUDY: "The Role of Cholesterol-lowering medications ('statins') in the progression of Age-related Macular Degeneration"

I………………………………………………………………………………….

hereby consent to **continue participating** in the clinical study evaluating the role of **statins** in the eye disease Age-related Macular degeneration.

I am aware that my participation in this study is voluntary and I am free to withdraw at any stage without this hindering any further medical treatment I may require.

I acknowledge that the aims, requirements, risks and side effects of this clinical trial as outlined on the "Patient Information" sheet, have been explained to me.

I hereby consent to having:

- my pupils dilated for the purpose of having my macula photographed
- undergoing non-invasive psychophysical testing (tests which subtly measure how well you can see)
- undergoing non-invasive cardiovascular assessment
- having a blood sample taken at each visit
- taking the study medication allocated to me.

I **DO/DO NOT** give permission for the study investigators to access my medical records should they require further information than I can provide regarding my medical history.

………………………………………………   ……………………

Signature of participant **Date**

……………………………………………….   …………………….

Signature of Investigator /Research Assistant Date

APPENDIX 5: Participant information

PARTICIPANT INFORMATION FOR PATIENTS WITH AMD IN THE CLINICAL STUDY:

"THE ROLE OF STATINS IN AGE-RELATED MACULAR DEGENERATION"

Background.
You have been invited to participate in a research study examining the role of the cholesterol-lowering medication-**statins** in the progression of Age-related macular degeneration (AMD). This study is a project run in collaboration with the Centre for Eye Research Australia (CERA) at the Royal Victorian Eye and Ear Hospital and the Department of Vascular Sciences & Medicine at the Dandenong Hospital.

The chief investigator is Dr. Robyn Guymer who is an ophthalmologist at CERA.

AMD is currently an irreversible disease of the eye whereby the area affected is located at the centre of the retina referred to as the macula. As the name implies the disease is degenerative and increases in frequency with age, especially in people over 65 years of age. The early stages of the disease is characterised by the presence of lipid deposits in the retina known as "drusen" that are not uncommon in people aged over 40 years.

The progression of AMD may eventually lead to the loss of central vision that results in everyday activities such as reading, driving, writing and recognition of facial features being impaired.

To date, there is very limited effective treatment available for the many people affected with AMD. Therefore, it is important to find some way of curbing the increasing number of people who are affected. Many studies have looked at risk factors such as antioxidant status, environmental factors and a family history of the disease in an attempt to understand the way the disease develops. However, the only consistent factors associated with the disease have been smoking and a genetic link.

There is clear evidence that AMD is an inherited disease, however the actual genes responsible remain elusive. Therefore, part of this study will involve examining participants to search for genes involved in AMD. One gene of current interest is the APOE gene that is involved in cholesterol metabolism.

Rationale for the study.
The data obtained from a population-based study that was recently completed in our department, showed for the first time that the progression of AMD might be slowed in people who had reported taking cholesterol-lowering medications. As a result of this finding we have embarked upon this current study.

Objective of the study.
This study is designed to assess the affect of cholesterol-lowering medications on the progression of AMD. The study is a randomised trial, which means that 50% of participants will receive the active medication and 50% will receive a placebo.
Neither you the participant, nor we the investigators, will be aware of which you will be receiving and this

will be the situation until the study is completed.
You will also be asked to participate in the cardiovascular component of the study that will assess the changes to your arteries over the first month whilst you are taking the study medication.

Possible side effects of statins.
Statins have been reported to be generally well tolerated in numerous studies however there are some side-effects that have been associated with this medication. These include: muscle pain and weakness, rash, mild and temporary headache and an increase in liver function. These side effects generally occur in only a small number of people taking such medication. For the purpose of this study, we will be monitoring reports of such effects to ensure your health is not jeopardized.

What the study involves for you as a participant.
As a participant in this study we would require you to attend Caulfield General Medical Centre and the Royal Victorian Eye and Ear Hospital (RVEEH).

Your eyes will be examined and questionnaires answered. The visual component will involve the assessment of your vision followed by the administering of drops that will dilate the pupils of your eyes. This will enable the ophthalmologist to examine the back of the eye where the macula is located and to take photographs of your eyes that will later be used to detect signs of AMD. We will also need to take a fasting blood sample from you to determine your cholesterol level and for DNA analyses.

You will undergo psychophysical tests (tests which measure subtly how well you can see). These tests are non-invasive. However, they will once again require the use of eye drops which will dilate your pupils.

Once all these examinations have been completed you will receive the study medication that you are to take for the duration of your participation in this study.

Four weeks after your initial examination, you will be required to undertake a blood test to assess any side effects of the study medication. Three months after the initial visit and annually thereafter we will require you to return to the study centres (Caulfield, RVEEH and VCO) for the duration of the study that will be 3 years. These appointments will be organised by the study staff.

We thank you for your time and assistance in helping us undertake this important research. Please let us know if you have any problems with transport. For more details or any questions please contact Dr Robyn Guymer or Ms Nicola Hunt on (03) 9929 8360.

APPENDIX 6: Adverse event form

ADVERSE EVENT FORM FOR CLINICAL STUDY:
"THE ROLE OF SIMVASTATIN IN EARLY AGE-RELATED MACULAR DEGENERTAION"

PARTICIPANT ID NUMBER_______________ DATE:____________

Description of symptoms:____
____
____
____
____
____

Date symptoms commenced: ___________

Date symptoms ceased:________________

Effect on study treatment:____

____

Was study treatment ceased permanently? YES NO

Follow-up required: YES NO

If YES outline follow-up required: ____
____

Resolved no sequelae Continuing

APPENDIX 7: ADVERSE EVENT FOLLOW-UP FORM

ADVERSE EVENT—FOLLOW-UP FORM FOR CLINICAL STUDY:
"THE ROLE OF SIMVASTATIN IN EARLY AGE-RELATED MACULAR DEGENERTAION"

PARTICIPANT ID NUMBER_______________ DATE:____________

Description of ongoing symptoms:_______________________________________
____
____
____
____
____

Date symptoms commenced: ___________

Date symptoms ceased:________________

Effect on study treatment:____

____

Was study treatment ceased permanently? YES NO

Follow-up required: YES NO

If YES outline follow-up required: ____
____

Resolved no sequelae Continuing

APPENDIX 8: Letter to participant on commencing medication

The study medication is either simvastatin, at a dose of 40mg, or placebo.

<u>Instruction</u>
Take two tablets in the evening with food. Avoid drinking grapefruit juice within 4 hours of taking the medication as this may interfere with absorption.

Severe side effects are rare, but seek medical advice promptly if you experience muscle pain, tenderness or weakness, dark urine, or yellowing of skin or the whites of your eyes.

Please give the enclosed "information for GP" to your local doctor. Please remind him/her that you are now in this trial, especially when he is about to prescribe any drug (particularly antibiotics), as there are a few but important interactions.

Please call Dr Luba Robman on 92766175, or Dr Robyn Guymer on 99298360 if you have any questions regarding the medication or the trial.

APPENDIX 9: Letter to GP on commencing medication

Dear doctor,

Your patient has been enrolled into the Age-Related Maculopathy Statin Study (ARMSS). This study looks at whether statin impacts on the rate of progression of age-related maculopathy (ARM). 50% of participants will be randomized to receive simivastatin 40mg, and 50% will be on an identical looking placebo.

Please be aware that simvastatin can have the following interactions drug interactions:

- Bile acid binding resins – reduce gastrointestinal absorption of statin. Give statin at least one hour before, or 4 hours after, bile acid binding resin.
- Clarithromycin, itraconazole, ketoconazole, erythromycin – increase risk of myopathy or rhabdomyolysis – stop statin temporarily, for the duration of antimicrobial treatment.
- Cyclosporin – increases risk of myopathy or rhabdomyolysis; use lowest effective statin dose, monitor for adverse effects, and measure creatinine kinase if indicated.
- Gemfibrozil – increases risk of myopathy and rhabdomyolysis; measure creatinine kinase 1 month after starting combined treatment with statin, then repeat at 6 month intervals and if symptoms of myopathy or rhabdomyolysis occur.
- Nicotinic acid (lipid lowering doses) – increases risk of myopathy and rhabdomyolysis; monitor clinically; measure creatinine kinase if indicated.
- Warfarin – statin may increase the INR and risk of bleeding; adjust warfarin dose according to INR.

Potential adverse effects:

- Common – myalgia, mild transient gastrointestinal symptoms, headache, insomnia, dizziness
- Rare – myopathy (with or without creatinine kinase elevation), rhabdomyolysis, renal failure, hepatitis, liver failure, alopecia, paraesthesia, peripheral neuropathy, impotence, nightmares, gynaecomastia, hypersensitivity, anaphylaxis, angioedema, toxic epidermal necrolysis, delayed wound healing
- Other – minor transient increases in transaminase and creatinine kinase are commonly found in the first weeks of treatment and appear to be dose related.

If there is any suspected side effect, or a clinical need to place your patient on a statin, or any of the medications mentioned above, we would very much appreciate you first contacting our chief investigator Robyn Guymer, or Dr Luba Robman on (03)99298360 before starting. Thank you for your cooperation.

APPENDIX 10. FLOW CHART FOR PROCEDURES INVOLVED IN THE ARMSS STUDY.

	Initial visit	1 month	4-6 months	12 months	24 months	36 months or “exit”
Study explanation	✔					
Consent	✔					
Randomisation	✔					
Fasting blood sample	✔	✔	✔	✔	✔	✔
Medical history	✔	✔	✔	✔	✔	✔
Listing of medications	✔	✔	✔	✔	✔	✔
Mini-mental questionnaire	✔					
IVI survey	✔					✔
Visual acuity and refraction	✔		✔	✔	✔	✔
Pupil dilation	✔		✔	✔	✔	✔
Retinal examination	✔		✔	✔	✔	✔
Macular photographs	✔		✔	✔	✔	✔
Blood pressure	✔		✔	✔	✔	✔
Psychophysics	✔		✔	✔	✔	✔
Reporting of side-effects		✔	✔	✔	✔	✔
